# Supplementary material for: Characterization of Martelella soudanensis sp. nov., Isolated from a Mine Sediment
Source: Microorganisms. 2021 Aug 14;9(8):1736. doi: 10.3390/microorganisms9081736 (PMC8401316; doi:10.3390/microorganisms9081736)
Supplement: Supplementary file 1 [file microorganisms-09-01736-s001.zip › microorganisms-1331130-supplementary.pdf]

Supplementary data

Characterization of *Martelella Soudanensis* sp. nov., Isolated from a Mine Sediment

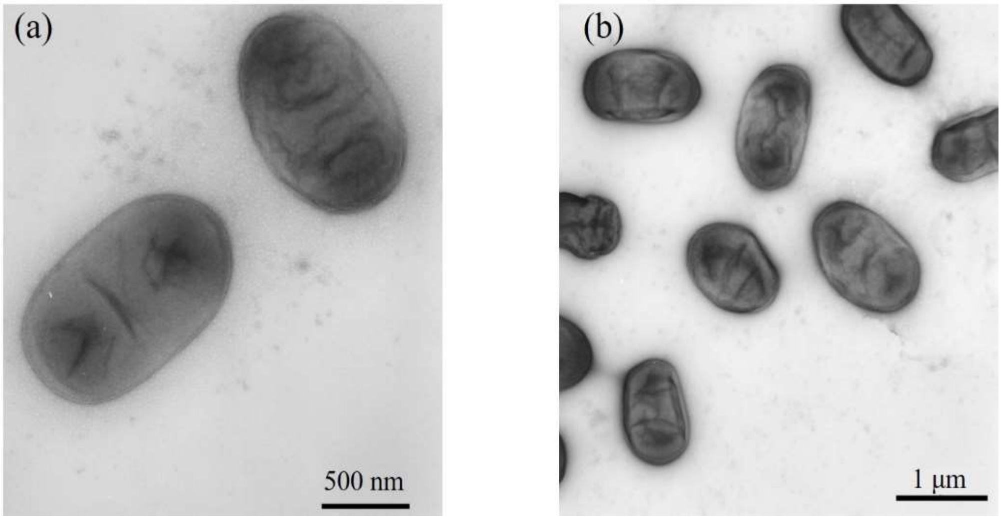

Supplementary Figure S1. Morphology of strains NC18^T (a) and NC20 (b) examined transmission electron microscope. Cells were grown on MA plate at 30℃ for 2 days.

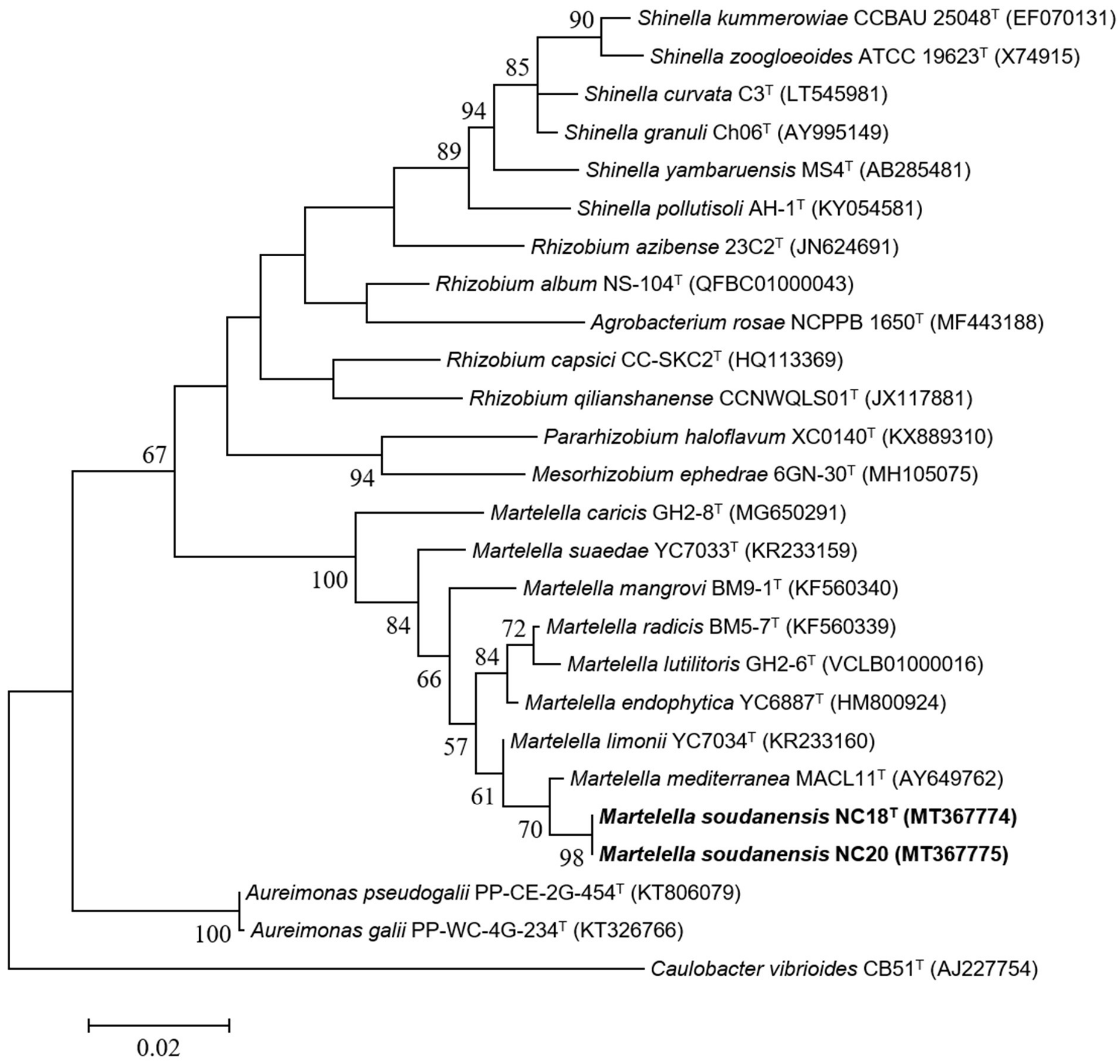

Supplementary Figure S2. Phylogenetic tree based on 16S rRNA gene sequences of strains NC18^T and NC20 with other related taxa using 1393 bp sequence. Evolutionary distances calculated using Jukes–Cantor model. Evolutionary history inferred using maximum-likelihood method. Bootstrap values (tested as 1000 replications) above 50% are shown next to the branches. The sequence of *Caulobacter vibrioides* CB51^T was used as outgroup. Bar, 0.02 nucleotide substitution per position.

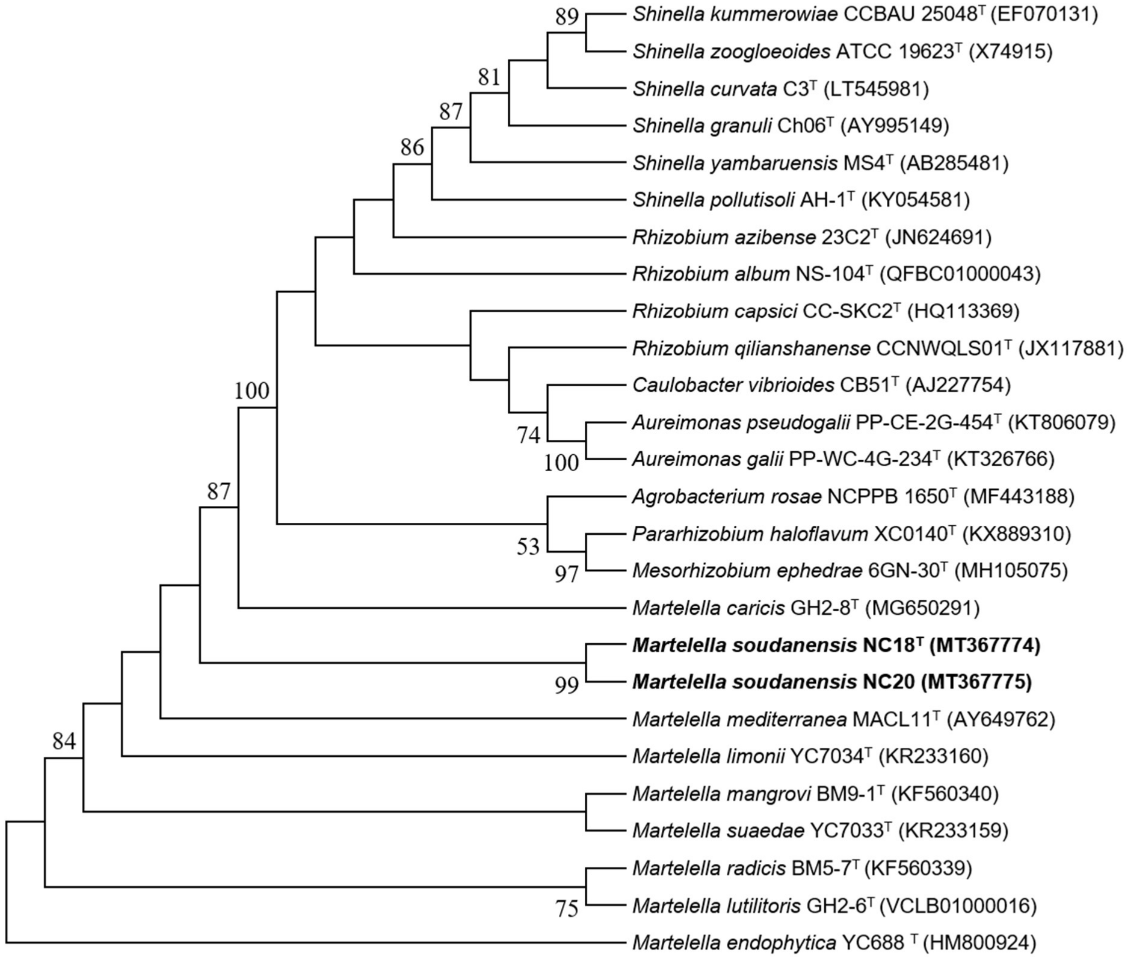

Supplementary Figure S3. Phylogenetic tree based on 16S rRNA gene sequences of strains NC18^T and NC20 with other related taxa using 1393 bp sequence. Evolutionary history inferred using maximum-parsimony method. Bootstrap values (tested as 1000 replications) above 50% are shown next to the branches.

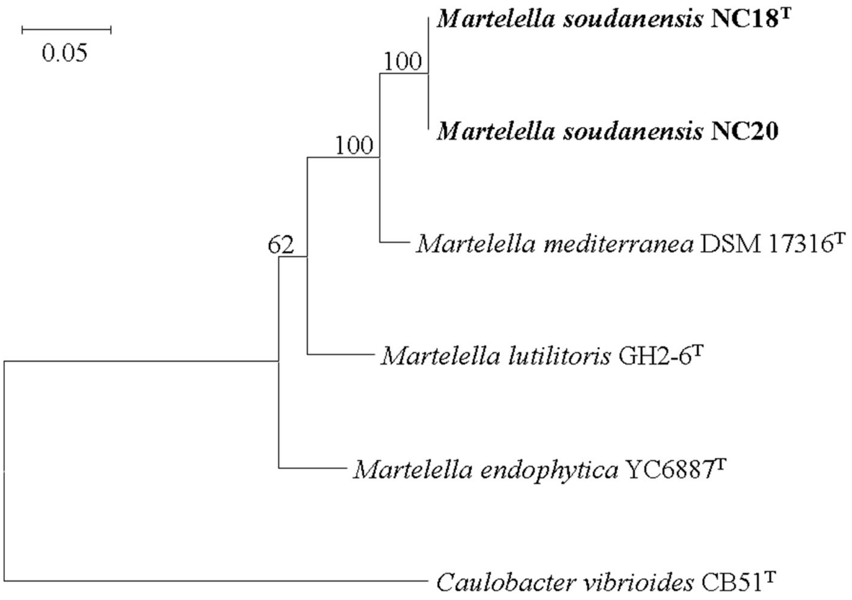

Supplementary Figure S4. Multilocus sequence analysis (MLSA) tree based on universally conserved protein sequences of strains NC18^T and NC20 with other related taxa. Evolutionary distances computed using JTT matrix-based method. Evolutionary history inferred using maximum-likelihood method. Bootstrap values (tested as 1000 replications) above 50% are shown next to the branches. The sequence of *Caulobacter vibrioides* CB51T was used as outgroup. Bar, 0.05 nucleotide substitution per position. The accession numbers for each sequence is shown in Supplementary Table S1.

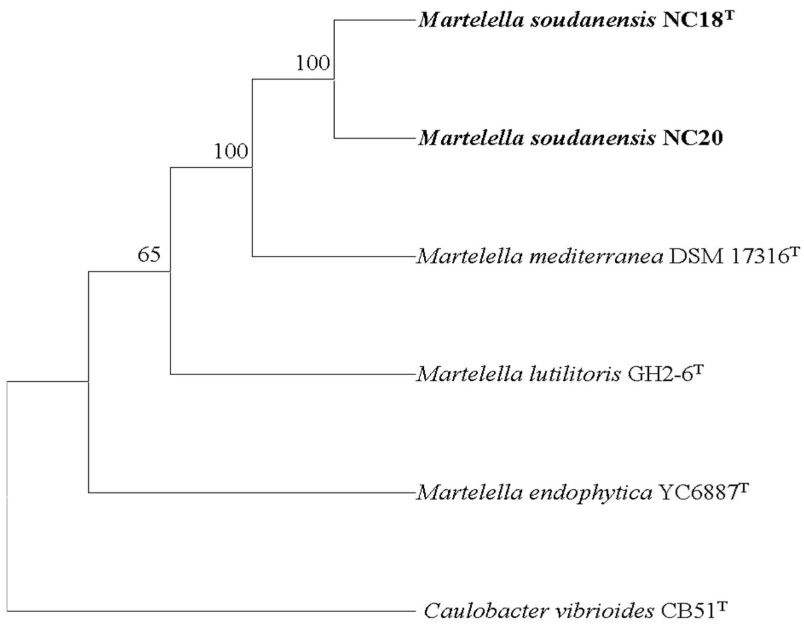

Supplementary Figure S5. Multilocus sequence analysis (MLSA) tree based on universally conserved protein sequences of strains NC18^T and NC20 with other related taxa. Evolutionary history inferred using maximum-parsimony method. Bootstrap values (tested as 1000 replications) above 50% are shown next to the branches. The accession numbers for each sequence is shown in Supplementary Table S1.

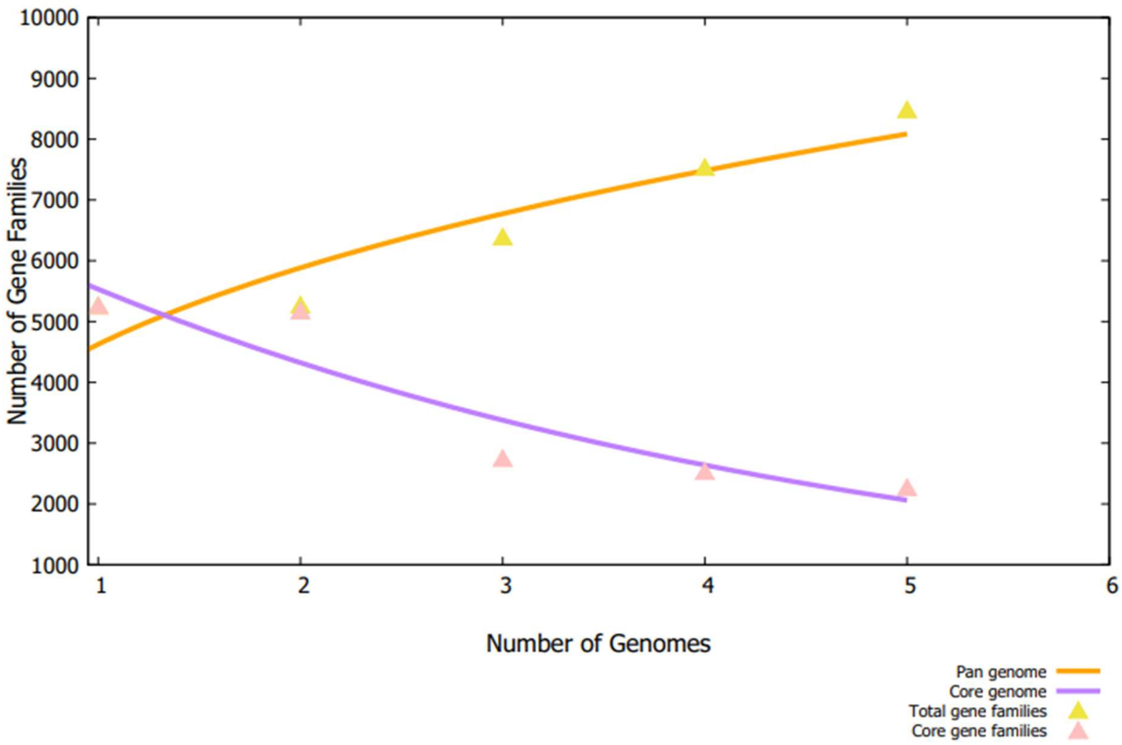

Supplementary Figure S6. Pan-genome curve of five *Martelella* strains. Analysis performed using the Bacterial Pan Genome Analysis Tool (BPGA) pipeline with default parameters. The pan-genome refers to the total number of orthologous gene families in the five *Martelella* strains; 2 isolated strains and 3 reference strains with available genome sequences.

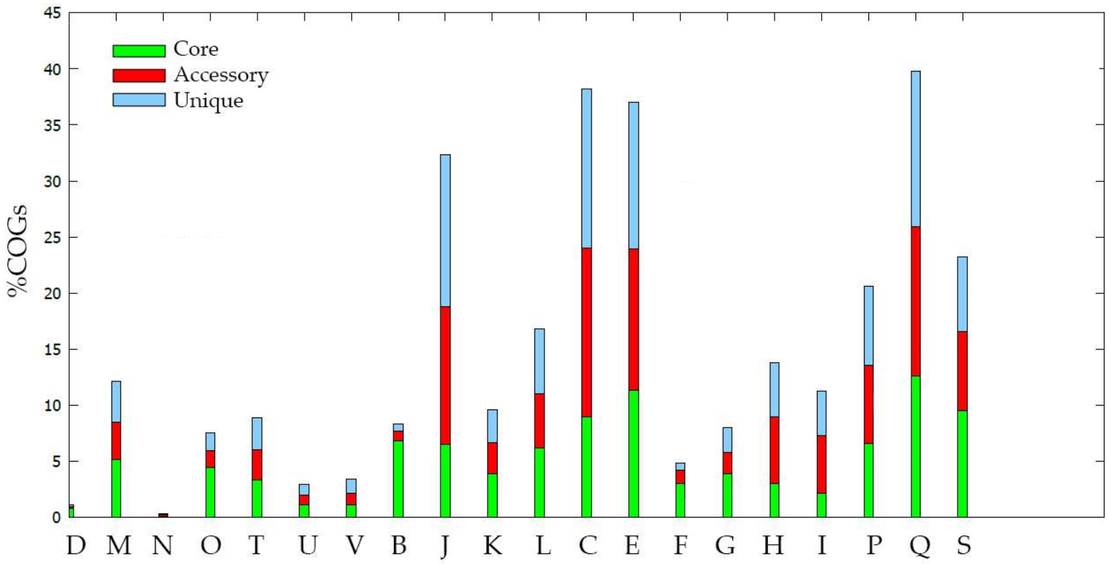

Supplementary Figure S7. Functional POGs annotation of five *Martelella* strains using COG database. One-letter abbreviations for the COG categories: D, cell cycle control, cell division, chromosome partitioning; M, cell wall/membrane/envelope biogenesis; N, cell motility; O, post-translational modification, protein turnover, and chaperones; T, signal transduction mechanisms; U, intracellular trafficking, secretion, and vesicular transport; V, defense mechanisms; B, chromatin structure and dynamics; J, translation, ribosomal structure and biogenesis; K, transcription; L, replication, recombination, and repair; C, energy production and conversion; E, amino acid transport and metabolism; F, nucleotide transport and metabolism; G, carbohydrate transport and metabolism; H, coenzyme transport and metabolism; I, lipid transport and metabolism; P, inorganic ion transport and metabolism; Q, secondary metabolites biosynthesis, transport, and catabolism; S, function unknown. Core, accessory, and unique genes represented as green, red, or blue bars, respectively.

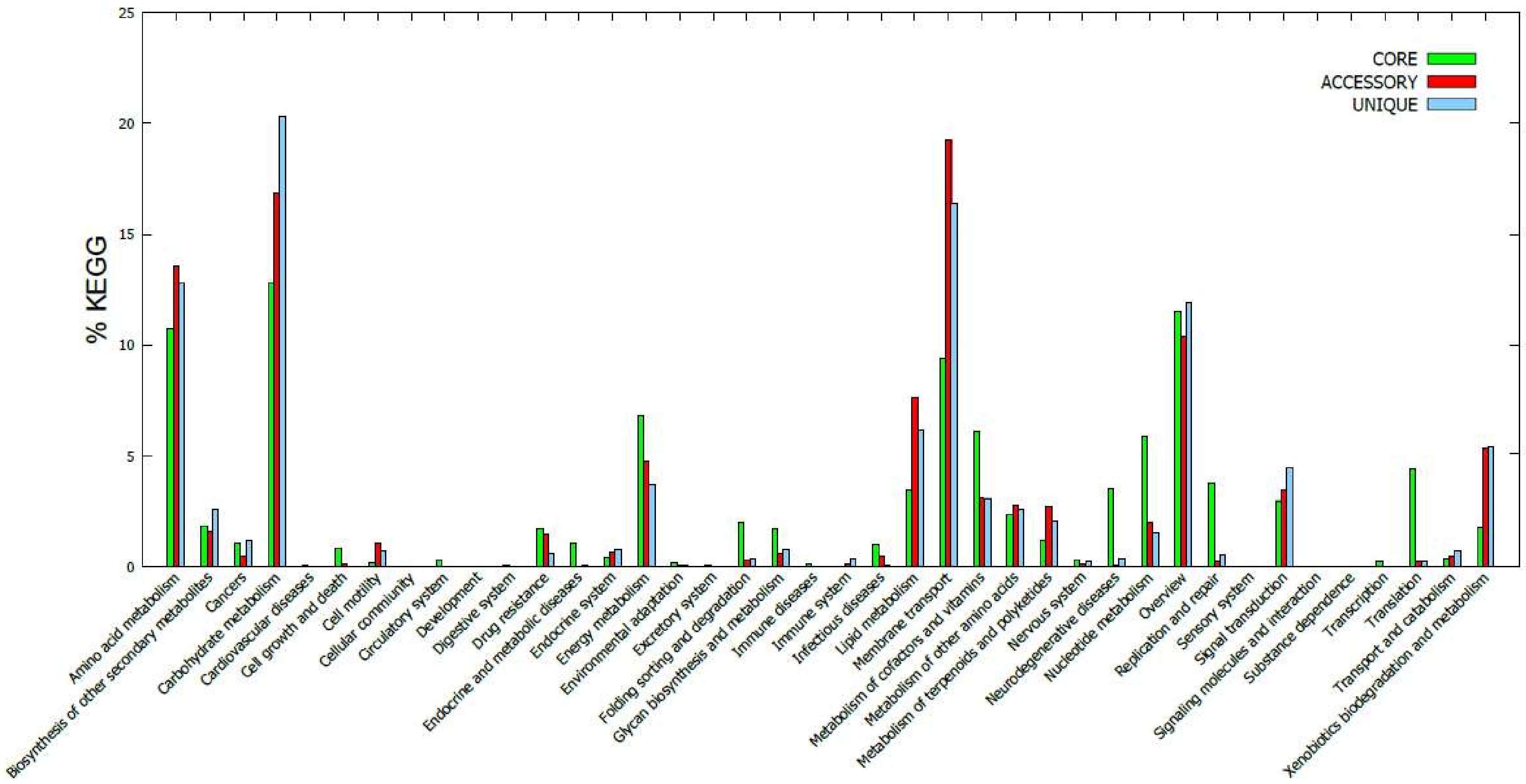

Supplementary Figure S8. Functional POGs annotation of five *Martelella* strains using KEGG database. Core, accessory, and unique genes represented as green, red, or blue bars, respectively.

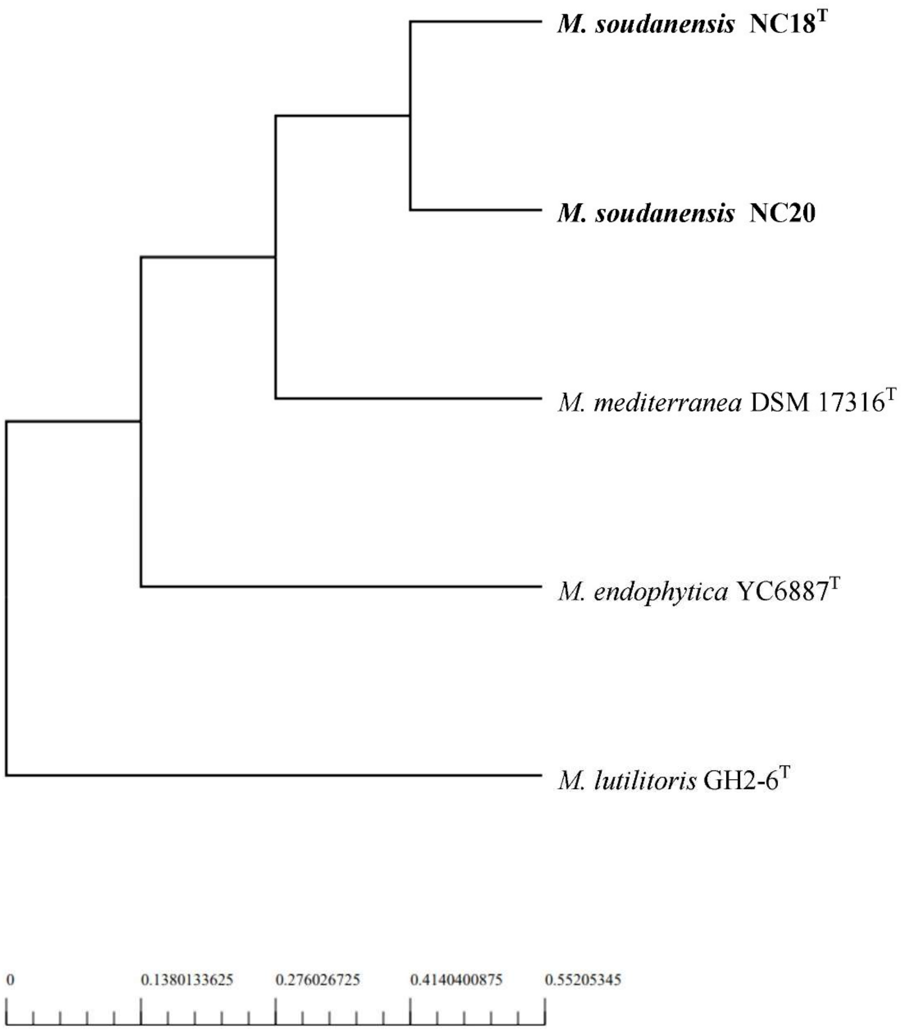

Supplementary Figure S9. Phylogenetic tree using concatenated POG core based on pan-matrix of five *Martelella* strains. The tree was constructed using the Neighbor-Joining method with 1,000 bootstrap replications.

Supplementary Table S1. Accession numbers of 31 universally conserved gene sequences used in multilocus sequence analysis (MLSA) tree.

	Strain					
	***M. soudanensis* NC18^{T}**	***M. soudanensis* NC20**	***M. mediterranea* DSM 17316^{T}**	***M. endophytica* YC6887^{T}**	***M. lutilitoris* GH2-6^{T}**	***Caulobacter vibrioides* CB51^{T}**
Accession No.						
dnaG	NZ_CP054858: 3247026-3245038	NZ_CP054861: 3181715-3183703	NZ_CP020330: 3698105-3696090	NZ_CP010803: 2360557-2362542	VCLB01000004: 80344-78320	PJRR01000004: 123475-123606
frr	NZ_CP054858: 2388168-2388767	NZ_CP054861: 4040639-4040040	NZ_CP020330: 3421289-3420687	NZ_CP010803: 3266682-3267302	VCLB01000009: 140281-139691	PJRR01000019: 318012-317149
infC	NZ_CP054858: 5086601-5085969	NZ_CP054861: 1342094-1342726	NZ_CP020330: 959918-959286	NZ_CP010803: 443173-442574	VCLB01000003: 385048-385707	PJRR01000017: 76299-76400
nusA	NZ_CP054858: 3858057-3856363	NZ_CP054861: 2570661-2572355	NZ_CP020330: 4381124-4379427	NZ_CP010803: 4533627-4531957	VCLB01000002: 386496-388199	PJRR01000012: 117603-117373
pgk	NZ_CP054858: 1842675-1841281	NZ_CP054861: 4586148-4587542	NZ_CP020330: 2526372-2525110	NZ_CP010803: 2825966-2824623	VCLB01000004: 141008-142357	PJRR01000016: 68752-68603
pyrG	NZ_CP054858: 3282298-3280547	NZ_CP054861: 3146441-3148192	NZ_CP020330: 3732770-3731121	NZ_CP010803: 3101104-3102780	VCLB01000008: 188632-186656	PJRR01000019: 125576-125683
rplA	NZ_CP054858: 2385066-2386145	NZ_CP054861: 4043741-4042662	NZ_CP020330: 3424362-3423310	NZ_CP010803: 3263603-3264652	VCLB01000004: 143165-142323	PJRR01000014: 320547-319738
rplB	NZ_CP054858: 1577953-1578813	NZ_CP054861: 4850878-4850018	NZ_CP020330: 4257348-4256488	NZ_CP010803: 4290064-4289204	VCLB01000005: 213914-214774	PJRR01000022: 11023-11307
rplC	NZ_CP054858: 1576225-1577025	NZ_CP054861: 4852606-4851806	NZ_CP020330: 4259031-4258276	NZ_CP010803: 4291695-4290991	VCLB01000005: 212216-212986	PJRR01000022: 9532-9708
rplD	NZ_CP054858: 1577010-1577645	NZ_CP054861: 4851821-4851186	NZ_CP020330: 4258291-4257656	NZ_CP010803: 4291006-4290371	VCLB01000005: 212971-213606	PJRR01000022: 10313-10492
rplE	NZ_CP054858: 1582175-1582732	NZ_CP054861: 4846656-4846099	NZ_CP020330: 4253128-4252571	NZ_CP010803: 4285839-4285282	VCLB01000005: 218139-218696	PJRR01000022: 14296-14853
rplF	NZ_CP054858: 1583506-1584057	NZ_CP054861: 4845325-4844774	NZ_CP020330: 4251798-4251247	NZ_CP010803: 4284509-4283958	VCLB01000005: 219469-22002	PJRR01000022: 15593-16141
rplK	NZ_CP054858: 1556127-1556603	NZ_CP054861: 4872704-4872228	NZ_CP020330: 4281403-4280930	NZ_CP010803: 4312090-4311647	VCLB01000004: 188532-188068	PJRR01000014: 121651-122142

	Strain					
	M. soudanensis **NC18^T**	*M. soudanensis* **NC20**	*M. mediterranea* **DSM 17316^T**	*M. endophytica* **YC6887^T**	*M. lutilitoris* **GH2-6^T**	*Caulobacter vibrioides* **CB51^T**
rplL	NZ_CP054858: 1558229-1558615	NZ_CP054861: 4870602-4870216	NZ_CP020330: 4279307-4278921	NZ_CP010803: 4310006-4309620	VCLB01000004: 186448-186062	PJRR01000014: 273210-272827
rplM	NZ_CP054858: 2609178-2608570	NZ_CP054861: 3819621-3820229	NZ_CP020330: 3226119-3226718	NZ_CP010803: 3374716-3374252	VCLB01000009: 128751-129224	PJRR01000007: 32280-32855
rplN	NZ_CP054858: 1581447-1581857	NZ_CP054861: 4847384-4846974	NZ_CP020330: 4253856-4253446	NZ_CP010803: 4286637-4286158	VCLB01000005: 217366-217821	PJRR01000022: 13621-13989
rplP	NZ_CP054858: 1580240-1580656	NZ_CP054861: 4848591-4848175	NZ_CP020330: 4255061-4254645	NZ_CP010803: 4287777-4287361	VCLB01000005: 216202-216618	PJRR01000022: 12722-13156
rplS	NZ_CP054858: 1188429-1188908	NZ_CP054861: 5240408-5239929	NZ_CP020330: 4531729-4531250	NZ_CP010803: 1975001-1975513	VCLB01000001: 152328-151822	PJRR01000012: 143754-144164
rplT	NZ_CP054858: 5085542-5085105	NZ_CP054861: 1343153-1343590	NZ_CP020330: 958840-958427	NZ_CP010803: 442063-441650	VCLB01000003: 386193-386612	PJRR01000017: 81262-81618
rpmA	NZ_CP054858: 4208949-4209230	NZ_CP054861: 2219762-2219481	NZ_CP020330: 1908584-1908303	NZ_CP010803: 73429-73710	VCLB01000001: 39867-40148	PJRR01000012: 22830-23105
rpoB	NZ_CP054858: 1558814-1562950	NZ_CP054861: 4870017-4865881	NZ_CP020330: 4278728-4274592	NZ_CP010803: 4309436-4305285	VCLB01000004: 185789-181638	PJRR01000014: 268628-264345
rpsB	NZ_CP054858: 2385066-2386145	NZ_CP054861: 4043741-4042662	NZ_CP020330: 3424362-3423310	NZ_CP010803: 3263603-3264652	VCLB01000009: 143165-142323	PJRR01000019: 320547-319738
rpsC	NZ_CP054858: 1579475-1580206	NZ_CP054861: 4849356-4848625	NZ_CP020330: 4255952-4255095	NZ_CP010803: 4288692-4287811	VCLB01000005: 215436-216167	PJRR01000022: 11953-12711
rpsE	NZ_CP054858: 1584428-1585165	NZ_CP054861: 4844403-4843666	NZ_CP020330: 4250767-4250144	NZ_CP010803: 4283497-4282820	VCLB01000005: 220410-221138	PJRR01000022: 16491-17111
rpsI	NZ_CP054858: 2608630-2608091	NZ_CP054861: 3820169-3820708	NZ_CP020330: 3226658-3227197	NZ_CP010803: 3374312-3373776	VCLB01000009: 129164-129703	PJRR01000007: 32859-33332
rpsJ	NZ_CP054858: 1575858-1576202	NZ_CP054861: 4852973-4852629	NZ_CP020330: 4259448-4259098	NZ_CP010803: 4292157-4291813	VCLB01000005: 211813-212157	PJRR01000022: 8263-8736
rpsK	NZ_CP054858: 1588870-1589346	NZ_CP054861: 4839961-4839485	NZ_CP020330: 4246540-4246064	NZ_CP010803: 4279265-4278807	VCLB01000005: 224798-225259	PJRR01000022: 20873-21262
rpsM	NZ_CP054858: 1588360-1588746	NZ_CP054861: 4840471-4840085	NZ_CP020330: 4247202-4246660	NZ_CP010803: 4279925-4279368	VCLB01000005: 224345-224731	PJRR01000022: 20470-20859
rpsS	NZ_CP054858: 1578832-1579107	NZ_CP054861: 4849999-4849724	NZ_CP020330: 4256475-4256194	NZ_CP010803: 4289185-4288910	VCLB01000005: 214787-215068	PJRR01000022: 11298-11576

	Strain					
	M. soudanensis **NC18^T**	*M. soudanensis* **NC20**	*M. mediterranea* **DSM 17316^T**	*M. endophytica* **YC6887^T**	*M. lutilitoris* **GH2-6^T**	*Caulobacter vibrioides* **CB51^T**
smpB	NZ_CP054858: 2247632-2248165	NZ_CP054861: 4181177-4180644	NZ_CP020330: 3309762-3309247	NZ_CP010803: 3155293-3155799	VCLB01000009: 42829-42260	PJRR01000021: 39502-39020
tsf	NZ_CP054858: 2386177-2387325	NZ_CP054861: 4042630-4041482	NZ_CP020330: 3423247-3422132	NZ_CP010803: 3264746-3265879	VCLB01000009: 142465-141119	PJRR01000019: 319610-318660

Supplementary Table S2. COG functional classification of the genome belonging to strains NC18^T and NC20.

Category letter	Function	The number of genes	
		NC18^T	NC20
	**CELLULAR PROCESSES AND SIGNALING**		
**D**	Cell cycle control, cell division, chromosome partitioning	22	22
**M**	Cell wall/membrane/envelope biogenesis	200	199
**N**	Cell motility	2	2
**O**	Post-translational modification, protein turnover, and chaperones	148	148
**T**	Signal transduction mechanisms	135	134
**U**	Intracellular trafficking, secretion, and vesicular transport	59	59
**V**	Defense mechanisms	90	89
	**INFORMATION STORAGE AND PROCESSING**		
**B**	Chromatin structure and dynamics	1	1
**J**	Translation, ribosomal structure, and biogenesis	173	173
**K**	Transcription	500	496
**L**	Replication, recombination, and repair	303	302
	**METABOLISM**		
**C**	Energy production and conversion	310	306
**E**	Amino acid transport and metabolism	537	526
**F**	Nucleotide transport and metabolism	102	99
**G**	Carbohydrate transport and metabolism	534	524
**H**	Coenzyme transport and metabolism	120	119
**I**	Lipid transport and metabolism	172	169
**P**	Inorganic ion transport and metabolism	458	451
**Q**	Secondary metabolites biosynthesis, transport, and catabolism	118	118
	**POORLY CHARACTERIZED**		
**R**	General function prediction only	1547	1530

Supplementary Table S3. Pan-genomes of five *Martelella* strains.

**Strain**	**No. of POG core**	**No. of POG accessory**	**No. of POG unique**
Martelella soudanensis NC18^T	2258	2906	9
Martelella soudanensis NC20	2258	2969	22
Martelella mediterranea DSM 17316^T	2258	1646	971
Martelella endophytica YC6887^T	2258	984	946
Martelella lutilitoris GH2-6^T	2258	994	647
